# Using Twitter Data Analysis to Understand the Perceptions, Beliefs, and Attitudes about Pharmacotherapy Used in Rheumatology: An Observational Study

**DOI:** 10.3390/healthcare11111526

**Published:** 2023-05-23

**Authors:** Adrian Abbasi-Perez, Miguel Angel Alvarez-Mon, Carolina Donat-Vargas, Miguel A. Ortega, Jorge Monserrat, Ana Perez-Gomez, Melchor Alvarez-Mon

**Affiliations:** 1 Service of Internal Medicine, Rheumatology and Autoimmune Diseases, University Hospital “Príncipe de Asturias”, 28805 Alcala de Henares, Spain; adrian.abbasi@salud.madrid.org (A.A.-P.); aperezalcala@yahoo.es (A.P.-G.); mademons@gmail.com (M.A.-M.); 2 Department of Medicine and Medical Specialities, Faculty of Medicine and Health Sciences, University of Alcala, 28805 Alcala de Henares, Spain; miguel.angel.ortega92@gmail.com (M.A.O.); jorge.monserrat@uah.es (J.M.); 3 Institute Ramon y Cajal for Health Research (IRYCIS), 28034 Madrid, Spain; 4 Cardiovascular and Nutritional Epidemiology, Institute of Environmental Medicine, Karolinska Institute, 17177 Stockholm, Sweden; carolina.donat.vargas@ki.se; 5 IMDEA-Food Institute, Campus of International Excellence, Universidad Autónoma de Madrid, Consejo Superior de Investigaciones Científicas, 28049 Madrid, Spain

**Keywords:** social media, rheumatology, methotrexate

## Abstract

Twitter has become an important platform for disseminating information about rheumatology drugs by patients, health professionals, institutions, and other users. The aim of this study was to analyze tweets related to 16 drugs used in rheumatology, including their volume, content, and type of user (patients, patients’ relatives, health professionals, health institutions, pharmaceutical industry, general press, scientific journals and patients’ associations), and to detect inappropriate medical content. A total of 8829 original tweets were obtained, with a random sample of 25% of the total number of tweets for each drug (at least 100 tweets) analyzed. Methotrexate (MTX) accounted for a quarter of all tweets, and there were significant differences in the proportion of tweets issued according to the type of user. Patients and their relatives mainly tweeted about MTX, while professionals, institutions, and patient associations posted more about TNF inhibitors. In contrast, the pharmaceutical industry focused on IL-17 inhibitors. Medical content prevailed in all drugs except anti-CD20 and IL-1 inhibitors and the most discussed medical topic was efficacy, followed by posology and adverse effects. Inappropriate or fake content was found to be very low. In conclusion, the majority of the tweets were about MTX, which is a first-line treatment for several diseases. The distribution of medical content varied according to the type of user. In contrast to other studies, the amount of medically inappropriate content was very low.

## 1. Introduction

By 2021, 93% of U.S. adults use the internet [1]. Around 70% of Americans use social media to connect with others, consume news, share information, and entertain themselves [2]. Twitter, which enables users to send short messages, no more than 280 characters in length, is one of the main social networks, used by 23% of Americans according to 2021 data [3]. 

Twitter is used by health providers and patients to discuss various health topics [4]. Studies have been conducted on health-related issues on Twitter, such as the role of healthcare workers in disseminating information [5], disease outbreak monitoring [6] and recruiting subjects for studies [7]. In addition, it has been shown how Twitter has become an increasingly popular social media platform among US physicians in recent years. Approximately 1 trillion tweets from 39,084 US physician accounts were analyzed to identify 6,399,146 tweets between 2016 and 2020 [8].

We are interested in rheumatology. Rheumatic diseases are a group of over 100 chronic illnesses that cause inflammation and loss of function in joints, tendons, ligaments, bones, and muscles. They affect more than 20% of US adults, with osteoarthritis, rheumatoid arthritis, spondylarthritis, gout and fibromyalgia being the most prevalent [9,10]. Patients often experience lifelong debilitating symptoms, reduced productivity at work, and high medical expenses [11]. Rheumatologists primarily use Twitter as a major intersection between free open-access medical education and social media. The American College of Rheumatology (ACR) Twitter account, @ACRheum, currently manages a list of more than 4000 rheumatologists and healthcare professionals interested in rheumatology [12,13]. Live-tweeting of curated information from medical conferences such as the ACR Annual Meeting or the European League Against Rheumatism (EULAR) enables the rapid sharing and dissemination of information to a worldwide audience. This practice also invites discussion with those who are unable to attend the meeting in person [14]. 

In addition to being a professional social network for healthcare providers, Twitter is also used by patients to exchange scientific information, share their personal experiences of the disease, and provide support to other patients, among other things [15].

A recent study analyzed the impact of rheumatic diseases on Twitter [16], while previous studies examined non-medical content on Twitter regarding biological treatments for inflammatory arthropathies [17]. However, to our knowledge, no study has been conducted to analyze all Twitter content related to drugs used in rheumatology, including inflammatory arthropathies and autoimmune diseases. It is crucial to address this knowledge gap so that healthcare professionals can act accordingly by analyzing the tweeted content.

We consider that drugs in rheumatology have an impact on the life of the patients, as these medications are often immunosuppressive. Furthermore, the use of these drugs can disrupt patients’ daily routines as it often requires regular medical follow-up, additional diagnostic tests, and the collection of medication from the hospital pharmacy.

However, the lack of analysis of data published on Twitter about these treatments has made it difficult to determine the interests of its users. As a result, we have decided to conduct an analysis of the content of tweets that mention medications used in rheumatology to gain a better understanding of the conversation around these drugs on social media. 

The aims of this study are the following: (i) Examine the volume and the kind of tweets related to treatments for rheumatic diseases. (ii) Describe the users who tweet and the main content of their tweets. (iii) Investigate which topics generate more tweets. (iv) Detect whether there is fake content (posts considered medically inappropriate according to the current medical knowledge). 

Given the importance of these drugs in rheumatology and the potential impact of social media on their use and perception, we believe that this study will provide valuable insights for healthcare professionals and patients alike.

## 2. Materials and Methods

### 2.1. Study Design and Data Source

In this observational quantitative and qualitative study, we focused on searching for tweets that referred to 16 rheumatic treatments. These drugs are methotrexate (MTX), tumor necrosis factor (TNF) blockers or anti-TNF (adalimumab, certolizumab, etanercept, golimumab, infliximab), costimulation B-cell activating factor blocker (abatacept), interleukin-12 (IL-12) and IL-23 inhibitor (ustekinumab), IL-17 inhibitors (secukinumab, ixekizumab), IL-6 inhibitors (sarilumab, tocilizumab), IL-1 inhibitors (Anakinra, canakinumab), anti-CD20 antibody (rituximab or RTX) and B-cell activating factor inhibitor or anti-Blys (belimumab). MTX is considered a conventional synthetic drug and all other drugs are considered biologic drugs.

Data were obtained in a period of four weeks, spanning from Monday, 25 January to Thursday, 20 February 2020. This time had a separation of at least two months from any major international rheumatology congress. The inclusion criteria for tweets were (1) being public (non-private); (2) being included in its content any of the drugs mentioned; (3) being posted in English or in Spanish and (4) being posted between 25 January and 20 February 2020.

### 2.2. Search Tool and Data Collection

In this work, we used the Twitter Firehose data stream, which is managed by Gnip and allows access to 100% of all public tweets that match a set of “search” criteria (query). 

In our study, the search criteria were the previously mentioned drugs. Tweet Binder, the search engine employed node.js and PHP language that enabled us to analyze tweets in a JavaScript Object Notation (JSON) format, which is used by Gnip.

### 2.3. Content Analysis Process and Creation of the Codebook

To assess the state of knowledge on the topic of interest, we performed a literature search on PubMed to review previous studies that analyzed the content of tweets in medicine, with a specific focus on rheumatology, and identified gaps in the field. Afterwards, we held several face-to-face meetings among the study participants to develop a comprehensive codebook for our analysis.

We then used Twitter Binder to obtain the tweets that included the word for one of the 16 drugs mentioned. A total of 8829 original tweets were obtained. For the study, a random sample of 25% for each drug out of the total number of tweets was selected. At least 100 tweets were analysed for each drug. This process led to the analysis of 1703 tweets of the 16 mentioned rheumatic drugs published on Twitter. Firstly, a codebook was specifically created based on our research questions, our previous experience in analyzing tweets and the most common tweet topics. 

Next, the 1703 selected tweets were scanned by two members of the research team. Each tweet was evaluated and classified as classifiable or unclassifiable based on its content. We obtained 1089 classifiable tweets using this method. Differences in categorization and other discrepancies between the raters were discussed with another author until a consensus was reached. Overall, the obtained reliability was higher than 90% for tweet content analysis. We considered a tweet as non-classifiable when its content did not provide enough information (for example, only contained nicknames of other users or an external link without accompanying comments) or was written in a language other than English or Spanish. For instance, many tweets containing the words “canakinumab” or “anakinra” as part of a username were non-classifiable. 

Supplementary Materials (Figure S1) shows a flowchart illustrating the process followed for the analysis of the tweets, including the number of included and excluded tweets. For classifiable tweets, we first analyzed the type of user. We distinguish among patients, family members of a patient, patient association, healthcare providers (doctors, nurses, physiotherapists, or researchers), institutions (official accounts of hospitals, universities, or scientific societies), pharmaceutical companies, scientific journals, and general information magazines.

Next, we analyzed whether tweets were related to medical or non-medical content, resulting in 636 tweets of medical content and 453 tweets of non-medical content. Among the tweets with medical content, we examined each tweet in detail. Specifically, we analyzed whether the tweet referred to the drug’s mechanism of action, pharmacokinetics, indication/posology, efficacy, comparison with another drug, or side effects. We also assessed whether the tweets contained web links to other pages, such as magazine articles or papers, and whether the linked content was scientifically rigorous or, conversely, lacked scientific support. In cases where the content lacked scientific rigor, we referred to it as fake content. This included tweets with erroneous content or content without scientific evidence, such as the use of herbal remedies for the cure of rheumatoid arthritis.

In our study, we also analyzed tweets with non-medical content, which we categorized into different topics depending on the subject of the tweet. These topics included commercial activity (such as advertising, purchase, and promotions), economics (related to drug prices, biosimilars, and medical coverage), disclosure for patients or training for healthcare professionals, personal opinions about the drug (positive or negative), requests for help, signs of support, and general comments that included the drug name. 

Supplementary Material (Table S1) provides a detailed description of the classification criteria we used, along with examples of tweets that fall into each category.

### 2.4. Ethical Considerations

This study received the approval of the University of Alcalá Research Ethics Committee and is compliant with the research ethics principles of the Declaration of Helsinki (seventh revision, 2013). This study did not directly involve human subjects, nor did it include any intervention; instead uses only publicly available tweets (subject to universal access through the internet according to the Terms of Service that all users on Twitter accept). Nevertheless, we have taken care to not reveal any in this report any username and to avoid citing tweets that could reveal usernames.

### 2.5. Statistical Analysis 

Data were analyzed to describe the frequencies of tweets and make comparisons between different characteristics of tweet content. The statistical difference between the number of tweets generated by characteristics of tweet content was calculated using Pearson’s chi-squared test, and statistical significance was set at a two-sided *p*-value < 0.05. These analyses were conducted with the software packages STATA v16 (StataCorp) and graphs using Microsoft Excel for Windows.

## 3. Results

### 3.1. Twitter Community Shows a Major Interest in the Most Prescribed Drugs

**Figure 1:**
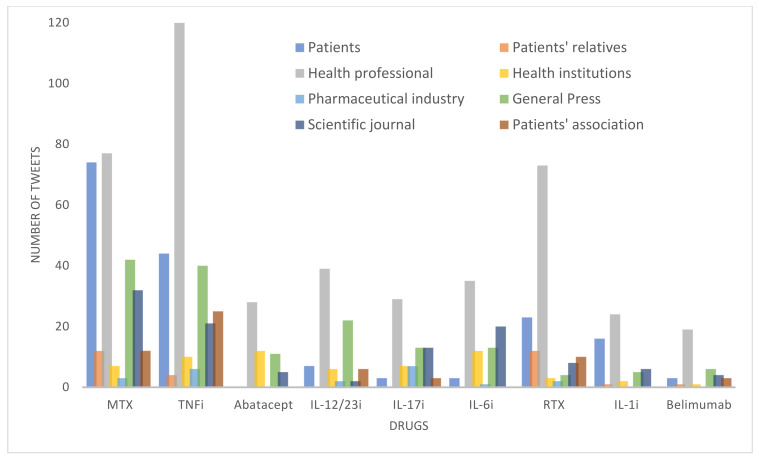
Distribution of tweets by type of user and drug mentioned. MTX, methotrexate; TNFi, tumor necrosis factor inhibitor; IL-12/23i, interleukin-12/23 inhibitor; IL-6i, interleukin-6 inhibitor; RTX, rituximab; IL-1i, interleukin-1 inhibitor.

We first analyzed the number of tweets generated by the main drugs used in rheumatology. The results, as shown in Table 1 and Figure 1, indicate that a significant proportion of tweets (24.7%) were related to a single drug, MTX, while TNF inhibitors accounted for 25.8% of the tweets, which included five different molecules. TNF inhibitors account for 25.8% of the tweets, but we must remember that this is a group of drugs that includes five molecules. In contrast, Anti-Blys (belimumab) only accumulated 3.4% of the tweets. The proportion of tweets issued by different user types showed statistically significant differences: patients and patient relatives posted tweets mostly about MTX, while professionals, healthcare institutions, and patient associations predominantly posted about TNF inhibitors. In contrast, the pharmaceutical industry focused its publications on IL-17 inhibitors. Scientific journals and the media distributed their publications more heterogeneously.

### 3.2. More Medical Content Is Tweeted Than Non-Medical Content and Fake Content Is Very Low

**Figure 2:**
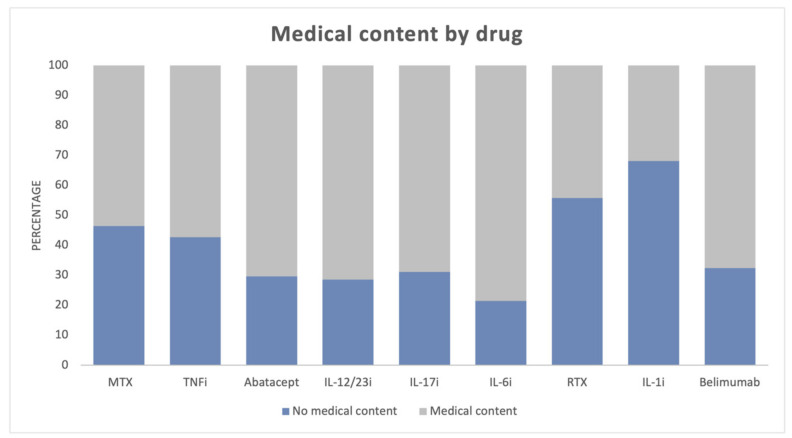
Proportion of medical and non-medical content among the different pharmacological groups. MTX, methotrexate; TNFi, tumor necrosis factor inhibitor; IL-12/23i, interleukin-12/23 inhibitor; IL-6i, interleukin-6 inhibitor; RTX, rituximab; IL-1i, interleukin-1 inhibitor.

Regarding the proportion of medical and non-medical content among the different pharmacological groups, we found statistically significant differences. Medical content prevailed in all pharmacological groups except for RTX and IL-1 inhibitors (Table 2 and Figure 2).

In addition, we analyzed the scientific accuracy of tweets with medical content and found statistically significant differences among the different pharmacological groups, with lower proportions of inaccurate content (Table 3). The analysis revealed that there were very few tweets with inappropriate or unscientific content. Only eight tweets (seven about MTX and one about an IL-6 inhibitor) contained inaccurate information. In contrast, when tweets referred to medical content, they were either referenced (59% in the case of MTX and 78% in the case of TNF inhibitors) or did not require bibliographic support. These eight tweets were posted by an unidentified user, two by healthcare professionals, and five by the general press.

### 3.3. The Distribution of Medical and Non-Medical Content Varies According to the Type of User

This study shows that the content of tweets varies depending on the user. Table 4 illustrates that healthcare personnel mostly post tweets with medical content (discussing mechanism of action, pharmacokinetics, or dosage), while patients and their relatives tend to post tweets with non-medical content (seeking support, complaining about the drug, etc.). Regarding non-medical content, we identified a predominant type of user depending on the topic. Patients were the most frequent users in tweets discussing economic issues, followed by healthcare professionals and pharmaceutical companies. Healthcare professionals and institutions were prominent in tweets related to dissemination. In tweets expressing personal opinions (Table S2, Supplementary Material), negative tweets were more common than positive ones (67.9% vs. 32.1%). In the case of MTX, most opinions were negative, while for the other treatments, opinions were equally distributed between positive and negative.

**Figure 3:**
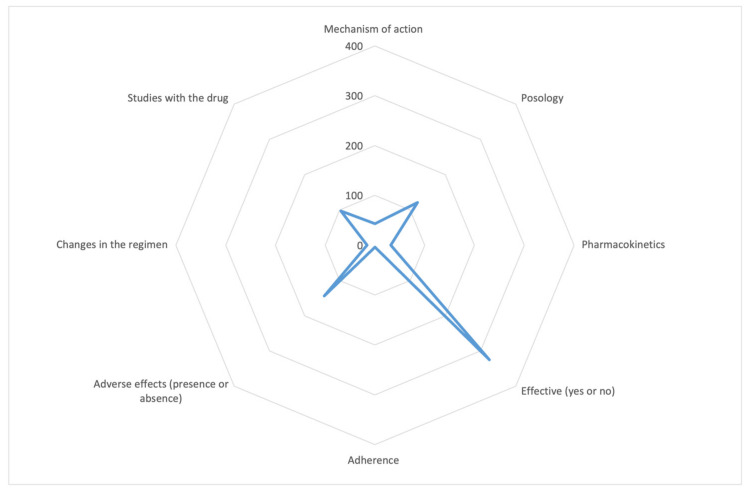
Distribution of tweets with medical content.

Regarding medical content, efficacy was the most discussed topic, followed by dosage and adverse effects (see Figure 3). Among tweets related to drug efficacy, 88.3% reported the drug as effective, while only 11.7% reported otherwise. Out of the 122 tweets discussing adverse effects, 85.5% described their presence, primarily authored by healthcare professionals.

Of the tweets discussing medical content, 66% cited a scientific publication, while 2.5% cited a non-scientific publication. A total of 31.5% of tweets did not include any references. 

**Figure 4:**
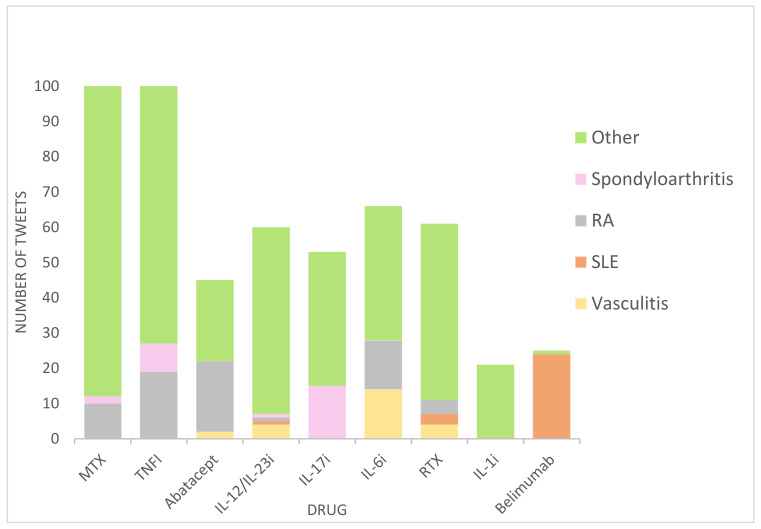
Distribution of tweets according to drug and disease. MTX, methotrexate; TNFi, tumor necrosis factor inhibitor; IL-12/23i, interleukin-12/23 inhibitor; IL-6i, interleukin-6 inhibitor; RTX, rituximab; IL-1i, interleukin-1 inhibitor; RA, rheumatoid arthritis; SLE, systemic lupus erythematosus.

Disease was mentioned in 70.3% of medical tweets. Figure 4 presents the data for those tweets where a disease is mentioned. Here, it can be seen that 10.6% were related to rheumatoid arthritis (RA), 4.4% to systemic lupus erythematosus (SLE), 4% to spondyloarthritis, and 3.7% to vasculitis. The remaining tweets concerned non-rheumatologic diseases, such as psoriasis (6.92%) or other medical conditions (40.4%). 

#### There Are Few Tweets about Adherence

The scarce content about adherence to treatment is remarkable, of which we found only four tweets (Figure 3). We will address this point in Section 4.

## 4. Discussion

In this study, our analysis revealed that the majority of tweets pertained to the most commonly prescribed drugs. Further examination by the drug group indicated that medical content was more prevalent than non-medical content. Notably, we found that nearly all of the tweets contained accurate information, with only a small number of tweets containing false information. Additionally, we observed that the type of content varied based on the type of user, with healthcare professionals primarily sharing medical content and patients sharing non-medical content. Of the medical content, efficacy was the most commonly tweeted topic. Among the non-medical content, healthcare professionals often posted requests for assistance. Firstly, our study provides evidence of significant interest in the treatment of rheumatic diseases among both healthcare professionals and patients and their families, as indicated by the high number of tweets.

Analyzing Twitter data has enabled clinicians, healthcare systems, and policymakers to optimize resources and improve patient outcomes [18]. In a recent study on social media usage, 8% of patients followed their rheumatologist, while 4% followed other healthcare providers or fellow rheumatology patients on Facebook or Twitter. The study revealed that 77% were willing to follow their physicians, and 16% were willing to follow other healthcare providers or rheumatology patients. These findings indicate patients’ preference for following their disease-specific doctor and emphasize the significant role of rheumatologists in social networks [19]. 

A recent study examined social media usage among rheumatologists in the Spondyloarthritis International Society, finding that 66% of respondents used social media for work-related purposes, including obtaining new web resources, interacting with international colleagues, and establishing a web presence. The top barriers to greater adoption of social networks were time investment, confidentiality, and security issues [20].

Another work evaluated the use of Twitter by patients with rheumatic diseases to gather their perspectives on disease symptoms and medication [15]. The data collected highlighted expressions of general pain, flares, and fatigue, which could be considered clinically relevant data. These data provide an opportunity for the medical community to better inform patients about their conditions.

A study of rheumatic disease patients found that the majority (90%) preferred obtaining information about their condition through face-to-face interactions with physicians, while a smaller proportion favored online connections [19]. Additionally, a study found that TV was ranked as the least popular source of medical information [21]. Considering these data and the results of our research, Twitter can be considered a good place to exchange information; besides, it allows online chatting with questions and answers in which other users can participate. The use of Twitter among patients with ankylosing spondylitis has also been studied, with 75% and 79% of patients preferring it for discussions in 2016 and 2019, respectively [22]. The use of health forums declined from 2016 to 2019, while Twitter became a popular platform for influencers with spondylitis. Analyzing this issue in rheumatoid arthritis, it has been observed that there is often a lack of knowledge about the disease, with the interesting finding that most study participants did not obtain any AR-related knowledge from mass media [23]. Interestingly, most study participants did not obtain any AR-related knowledge from mass media. The authors suggest enhancing health programs through mass media to improve patients’ disease awareness, which is a conclusion also reached in a similar study [24]. All these data demonstrate the importance of Twitter as a tool to share information of interest to patients and healthcare professionals.

However, in a survey, rheumatologists indicated a preference for contacting patients through WhatsApp (36%), Facebook (27%), and rheumatology clinic websites/forums (25%), with no preference for Twitter [23]. Another paper in a different medium, however, has determined that Twitter was the most frequently used platform for professional development and was viewed as the most useful for improving knowledge, problem-solving, creativity, and other domains related to professional development [25]. 

In a survey of the Emerging European League Against Rheumatism Network (EMUNET) group, researchers discovered that rheumatologists and basic scientists extensively utilized social media for both social and professional purposes. The survey emphasized the importance of offering learning resources and promoting awareness of social media usage, which could enhance communication, participation, and collaboration, thus encouraging professional use [26]. Previous studies support our data, which demonstrate that the majority of tweets come from healthcare professionals [8,12].

Regarding drug analysis, we first analyzed MTX, which is recommended as the first-line therapy for RA by EULAR, and is the most frequently used conventional synthetic drug [27,28,29]. However, the drug’s efficacy relies on various factors, including treatment adherence. A recent study concluded that patients require more information on MTX’s interactions and adverse effects, as the information provided in the drug package insert was deemed insufficient [30]. Patients’ fear of the drug’s toxicity may cause them to discontinue treatment, highlighting the importance of open communication with a rheumatologist to modify patients’ knowledge and perceptions about the medication [31].

Studies have shown that while physicians receive positive feedback on MTX, most negative experiences are shared on social networks and internet forums [32], a result also found in our work. 

Scientific journals, accounting for almost 10% of tweets, surprisingly tweet more about drugs that have been available in the market for years, such as TNF inhibitors. This may be because recent drug experiments are often compared to drugs with established experience [33,34]. Other studies have also reported similar findings where drugs that are frequently used and have been on the market for a longer period of time accumulate more tweets [35]. The disparity in tweet frequency can also be attributed to the marketing of biosimilar drugs, currently only available in the TNF inhibitors group, which has recently gained traction [36,37].

Most RA patients, including those who have not used MTX before, prefer triple therapy with MTX as the initial treatment due to the higher probability of symptom improvement, even with an additional pill burden [38,39]. Studies have shown that patients with recent RA can be divided into two equally distributed profiles: those who prioritize symptom improvement and treatment benefits (54%) and those who are concerned about adverse effects. Some patients may prefer alternative effective options instead of anti-TNF therapy due to its potential to increase the risk of infections and malignant disease [40]. Out of all the collected tweets, 120 specifically refer to adverse effects (mostly from health professionals), 53 to negative experiences with the drug, and 25 to positive experiences (both from patients).

Next, we analyze biological drugs. All drugs discussed in our study are biological drugs, except for MTX. In a multicenter study conducted in South Korea, 80.1% of 139 patients with RA and 77.1% of 168 patients with spondyloarthritis reported being satisfied or very satisfied with the therapeutic effects of their current biological treatment. A total of 86.6% of the patients cited their physician as the primary source of information on biological treatment [41]. These findings are significant as the vast majority of patients are referred to their rheumatologist. This may partly explain the low number of tweets about biological treatment among patients. 

The ability to prevent joint damage was a significant factor for patients when deciding between TNF and non-TNF biologic drugs. Patients preferred more effective medications, and achieving remission was an essential consideration in treatment selection [42]. In addition, the therapeutic guidelines for rheumatoid arthritis and psoriatic arthritis recommend anti-TNF as the initial biologic treatment. If this treatment fails, a switch to a biologic other than anti-TNF may be considered, depending on the patient [43,44]. The prioritization of anti-TNF among biologic drugs may explain why it is the most frequently discussed group of biological drugs on Twitter.

The lack of tweets related to treatment adherence is noteworthy and is also reflected in the scientific literature. While there are studies assessing adherence in patients with conditions such as hypertension, diabetes, and epilepsy [45], there is a dearth of robust data on adherence in autoimmune diseases [46,47]. This is similarly observed in studies on the treatment of mental illness, where tweets related to adherence are infrequent or absent [35,48]. This lack of information is concerning as it means that we are missing out on valuable insights into patient behavior regarding treatment adherence both in research and in real-life settings via Twitter messages.

Regarding the type of tweets according to content, there is a higher number of tweets with medical content for all drugs, except for RTX and Anakinra. The overall safety profile of the studied drugs is satisfactory, although there is evidence suggesting a higher incidence of viral infections associated with RTX, even in monotherapy, compared to other rheumatology drugs [49]. This has been confirmed during the COVID-19 pandemic, which has shown increased mortality rates in RTX-treated patients with SARS-CoV-2 infection [50]. As for Anakinra, the lower number of medical tweets can be attributed to a single active user who shared mostly non-medical content regarding their personal experience with the treatment.

An important point we analyzed is the number of fake tweets. The high percentage of tweets written by healthcare professionals lends credibility to the content. This is in contrast to previous studies, where the incidence of fake tweets was significantly higher [16] and underscores the quality of the tweets related to our study topic. Other studies, where the tweets were mainly authored by non-healthcare users, showed a significant amount of fake content [51,52]. 

In terms of the most tweeted diseases, RA stands out as the most frequently mentioned rheumatic condition in our study. This could be attributed to the prevalence and the common utilization of treatments such as MTX or biologics [53,54]. As such, it is not surprising that healthcare professionals, the press, and scientific journals mention RA more often than other rheumatic diagnoses. However, patients, their relatives, and associations have limited tweets with medical content, which are mainly focused on non-medical topics.

Regarding adverse effects, health experts and scientific publications often refer to them, making up 18% of medical tweets. While conventional methods detect only 5% of treatment-related negative reactions, social media posts disclose up to 62% [55], showing interest in the topic. Drug concerns mainly relate to possible side effects [56] particularly cancer risk (40.1%) and tuberculosis activation risk (30.7%) as identified in the tweets analyzed.

This study has some limitations worth mentioning. Firstly, the design of the codebook and text analysis involves a certain degree of subjectivity, which may pose a challenge to its reproducibility. Secondly, the study did not analyze the commercial names of drugs, which could exclude an unknown number of relevant tweets that should be considered in future investigations. Thirdly, not all drugs used in rheumatology were included in the analysis, such as azathioprine, hydroxychloroquine, leflunomide, mycophenolate, and the emerging JAK inhibitors, among others. Fourthly, for canakinumab, 92% of tweets were deemed unclassifiable due to users whose usernames included the drug name but were not related to medication content.

Despite these limitations, we minimized their effects through robust training and a comprehensive codebook design. Moreover, the analysis was performed by rheumatologists.

## 5. Conclusions

In conclusion, our findings demonstrate a significant interest among Twitter users in the drugs utilized in rheumatology, including patients, relatives, healthcare professionals, and the pharmaceutical industry. The interests of each group differ, with medical content being more prevalent among healthcare professionals and non-medical content being more prevalent among patients. Encouragingly, there is limited fake content, which reinforces the notion that Twitter has evolved into a platform for medical professionals.

## Figures and Tables

**Table 1:** Drugs by type of user.

	MTX	TNFi	Abata-cept	IL-12/23i	IL-17i	Anti-IL 6	RTX	IL-1i	Belimu-mab	TOTAL
Undetermined	10	10	8	0	2	0	3	0	0	33
Patient	74	44	0	7	3	3	23	16	3	173
Patients’ relatives	12	4	0	0	0	0	12	1	1	30
Health professional	77	121	28	39	29	35	73	24	19	445
Health institution	7	10	12	6	7	12	3	2	1	60
Pharmaceutical industry	3	6	0	2	7	1	2	0	0	21
General press	42	40	11	22	13	13	4	5	6	156
Scientific journal	32	21	5	2	13	20	8	6	4	111
Patients’ association	12	25	0	6	3	0	10	1	3	60
Total	269	281	64	84	77	84	138	55	37	1.089

MTX, methotrexate; TNFi, tumor necrosis factor inhibitor; IL-12/23i, interleukin-12/23 inhibitor; IL-6i, interleukin-6 inhibitor; RTX, rituximab; IL-1i, interleukin-1 inhibitor.

**Table 2:** Proportion of medical and non-medical content among the different pharmacological groups.

(%)	MTX	TNFi	Abatacept	IL-12/23i	IL-17i	Anti-IL 6	RTX	IL-1i	Belimumab
Non-medical content	46.47	42.7	29.69	28.57	31.17	21.43	55.8	68.09	32.43
Medical content	53.53	57.3	70.31	71.43	68.83	78.57	44.2	31.91	67.57

MTX, methotrexate; TNFi, tumor necrosis factor inhibitor; IL-12/23i, interleukin-12/23 inhibitor; IL-6i, interleukin-6 inhibitor; RTX, rituximab; IL-1i, interleukin-1 inhibitor.

**Table 3:** Distribution of tweets containing medical information by drug, according to if they provide scientific literature or not, and if it is fake.

%	MTX	TNFi	Abatacept	IL-12/23i	IL-17i	Anti-IL 6	RTX	IL-1i	Belimumab	TOTAL
Not applicable	35.42	20.5	35.56	25	5.66	6.06	8.2	23.8	8	21.07
Scientific literature	59.72	79.95	64.44	75	94.34	92.42	91.8	76.1	92	77.68
Fake	4.86	0	0	0	0	1.52	0	0	0	1.30

MTX, methotrexate; TNFi, tumor necrosis factor inhibitor; IL-12/23i, interleukin-12/23 inhibitor; IL-6i, interleukin-6 inhibitor; RTX, rituximab; IL-1i, interleukin-1 inhibitor.

**Table 4:** Distribution of tweets according to the user and whether the content is medical or not.

User	No Medical Content, *n* (%)	Medical Content *n* (%)	TOTAL *n* (%)
Undetermined	27 (81.82)	6 (18.18)	33 (100)
Patient	165 (95.38)	8 (4.62)	173 (100)
Patients’ relatives	29 (96.67)	1 (3.33)	30 (100)
Health professional	145 (32.58)	300 (67.42)	445 (100)
Health institution	17 (28.33)	43 (71.67)	60 (100)
Pharmaceutical industry	13 (61.9)	8 (38.1)	21 (100)
General press	20 (12.82)	136 (87.18)	156 (100)
Scientific journal	3 (2.7)	108 (97.3)	111 (100)
Patients’ association	34 (56.67)	26 (43.33)	60 (100)
Total	453 (41.6)	636 (58.4)	1.089 (100)

## Data Availability

The data presented in this study are available on request from the corresponding author.

## Supplementary Materials

The following supporting information can be downloaded at: https://www.mdpi.com/article/10.3390/healthcare11111526/s1, Figure S1, Tables S1 and S2.
