# Supplementary material for: Using Twitter Data Analysis to Understand the Perceptions, Beliefs, and Attitudes about Pharmacotherapy Used in Rheumatology: An Observational Study
Source: Healthcare (Basel). 2023 May 23;11(11):1526. doi: 10.3390/healthcare11111526 (PMC10252953; doi:10.3390/healthcare11111526)
Supplement: Supplementary file 1 [file healthcare-11-01526-s001.zip › healthcare-2316125-supplementary.pdf]

Supplementary material

Table S1: Examples of tweets by category.

Categories	Examples of classification
**Unclassifiable**	@MALSWAR انه يبدوAdalimumab<br>و بمجرد تعرضه لدرجة حرارة الغرفة، يجب استخدامه خلال ١٤ يوم
**Classifiable**	
**Medical content**	
Mechanism of action	Methotrexate competitively inhibits dihydrofolate reductase (DHFR). #usmle
Indication of treatment or posology	The European Commission Approves Expanded Use of Janssens STELARA ustekinumab for the Treatment of Paediatric Patients With Moderate to Severe Plaque Psoriasis: The expanded use of ustekinumab addresses a high unmet need among children with moderate to… https://t.co/GOjiWP5TGP
Pharmacokinetics	GSK Phase 1 Single Dose Study to Investigate the Pharmacokinetics (PK) and Safety of Belimumab 200 Milligrams (mg) Intravenous and 200 mg Subcutaneous Via Auto-injector in Chinese Healthy Subjects…NCT04136145. Estimated Primary Completion Date - 03/2020
Side effects	ENTRACTE trial studied the risk of major adverse CV events (MACE) in 3080 RA pts Rx with tocilizumab (TCZ) vs etanercept (ETN), and found no increased risk of MACE in TCZ patients compared to ETN (HR 1.05 (95% con dence interval 0.77–1.43). https://t.co/QxOlzfDcZD
Efficacy	3 year Secukinumab data in ankylosing spondylitis, the 81% retention rate seems high, higher than what I would have expected as this is commonly being used 2nd line. https://t.co/F3cpyY3bER
Links to scientific references	In this #JAAPOS case report on acquired Brown syndrome, Adalimumab was initiated and led to complete resolution of the disease. https://t.co/mTC7LSR9xC
Links to non-scientific references	Research has shown that the Chinese herbal remedy Triptergium wilfordii Hook F (TwHF) functions as effectively as the standard drug treatment methotrexate, which is typically prescribed to deal with the symptoms of rheumatoid arthritis. https://t.co/PHFnjuIzJX
Administration schedule other than recommended (by patient or physician)	Loss of RESponse to Ustekinumab Treated by Dose Escalation: The aim of the study is to investigate the effect of reinduction with ustekinumab 6mgkg IV followed by two different maintenance dosing regimens 90 mg subcutaneous every 8 weeks Q8W vs 90 mg… https://t.co/s43WF18BVx
Comparison of the drug	Open label study of Abatacept in 79 patients with primary Sjogrens syndrome showed no difference between those

with other drugs	treated wtih ABA vs Placebo. https://t.co/tUfAn40k9S
**Non-medical content**	
Commercial activity	@xxx Interested in your perspective of the market opportunity of a high concentration adalimumab (i.e., CT-P17, AVT02). Offers a level of differentiation but still a late entrant to a crowded market
Economy	People w/ RA stable on etanercept (Enbrel) for 6+ mo. who experienced copay ⬆ > $40 were more likely to switch to another drug (p=0.021), less likely to be adherent, and less likely to be effectively treated 12 mo. later than those w/ no copay change. https://t.co/SdASoJeqCf
Training/dissemination	QD Clinic - videos and podcast on patient care issues - this week: isolated inflammatory hip arthritis; monitoring vasculitis; mistakes in pain management; when to use anakinra. Watch videos on https://t.co/V10S4oVFsv or listen by podcast https://t.co/8ryHEbAPXe
Personal opinions	
Positive	@xxx My mum has rituximab for RA and it's changed her life. I'm on the evoclumab for inherited high cholesterol, also life changing. I've done lots of clinical research in biologicals in all sorts of conditions and generally if you have a response they are life changing. Good luck
Negative	to explain to people that i've been going through something like chemotherapy (in fact, infliximab was intended for chemotherapy it's a d*** blck label drug) since 2016 is tiring
Comment including drug name	@xxx Not sure! Maybe depending on profiles? High IFN = ANIFRO, high IL12/23 or maybe low IFN = Ustekinumab or Bari.
Request for help	@xxx @xxx I read that coronovirus causes a cytokine storm in it's final stages which is responsible for most deaths. is that true? Could Anakinra, the anti-inflamatory drug be used to prevent deaths? https://t.co/obmHqGubba #coronavirus
Show of support	@xxx Hope your feeling better soon, don't know what meds your on but I got put on infliximab infusions 6 months ago, made a huge difference.

Figure S1: Flowchart of data management and content analysis

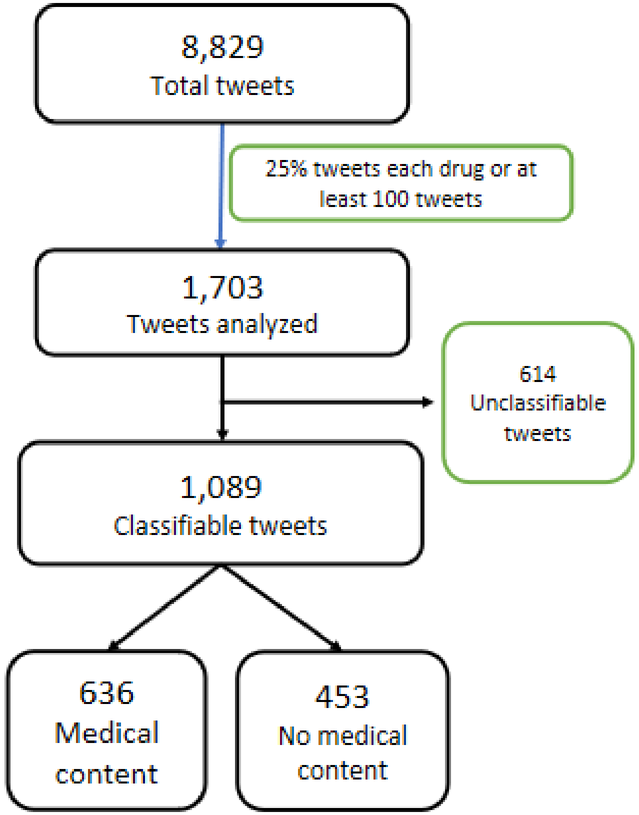

Table S2: number of tweets with content about drug opinion

	MTX	Others drugs	Total
Positive	10	15	25 (32.1%)
Negative	40	13	53 (67.9%)
Total	50	28	78 (100%)

MTX: methotrexate
